# An Integrated Platform for Serological Detection and Vaccination of COVID-19

**DOI:** 10.3389/fimmu.2021.771011

**Published:** 2021-12-23

**Authors:** Sung-Chan Wei, Wei-Ting Hsu, Chun-Hsiang Chiu, Feng-Yee Chang, Huei-Ru Lo, Chuan-Yu Liao, Hwai-I Yang, Yu-Chi Chou, Chih-Hsuan Tsai, Yu-Chan Chao

**Affiliations:** ^1^ Institute of Molecular Biology, Academia Sinica, Taipei, Taiwan; ^2^ Division of Infectious Disease and Tropical Medicine, Department of Internal Medicine, Tri-Service General Hospital, National Defense Medical Center, Taipei, Taiwan; ^3^ Genomics Research Center, Academia Sinica, Taipei, Taiwan; ^4^ Biomedical Translation Research Center, Academia Sinica, Taipei, Taiwan; ^5^ Department of Entomology, College of Agriculture and Nature Resources, National Chung Hsing University, Taichung, Taiwan; ^6^ Department of Entomology, College of Bioresources and Agriculture, National Taiwan University, Taipei, Taiwan; ^7^ Department of Plant Pathology and Microbiology, College of Bioresources and Agriculture, National Taiwan University, Taipei, Taiwan

**Keywords:** baculovirus, COVID-19, SARS-CoV-2, serological detection, vaccine

## Abstract

Coronavirus Disease 2019 (COVID-19), caused by Severe Acute Respiratory Syndrome Coronavirus 2 (SARS-CoV-2), is an ongoing pandemic. Detection and vaccination are essential for disease control, but they are distinct and complex operations that require significant improvements. Here, we developed an integrated detection and vaccination system to greatly simplify these efforts. We constructed recombinant baculoviruses to separately display the nucleocapsid (N) and spike (S) proteins of SARS-CoV-2. Insect cells infected by the recombinant baculoviruses were used to generate a cell-based system to accurately detect patient serum. Notably, although well-recognized by our newly developed detection system in which S-displaying insect cells acted as antigen, anti-S antibodies from many patients were barely detectable by Western blot, evidencing that COVID-19 patients primarily produce conformation-dependent anti-S antibodies. Furthermore, the same baculovirus constructs can display N (N-Bac) or S (S-Bac) on the baculovirus envelope and serve as vector vaccines. Animal experiments show that S-Bac or N-Bac immunization in mice elicited a strong and specific antibody response, and S-Bac in particular stimulated effective neutralizing antibodies without the need for adjuvant. Our integrated system maintains antigen conformation and membrane structure to facilitate serum detection and antibody stimulation. Thus, compared with currently available technologies, our system represents a simplified and efficient platform for better SARS-CoV-2 detection and vaccination.

## Introduction

Coronavirus Disease 2019 (COVID-19) caused by Severe Acute Respiratory Syndrome Coronavirus 2 (SARS-CoV-2) (1) has caused a lethal pandemic worldwide. As of July 2021, there have been more than 187 million confirmed cases, representing yet another coronavirus causing large-scale human infection after SARS-CoV and MERS-CoV (1). The emergence of new virus variants renders disease control a long-term challenge. Continuous monitoring of the immune status of individuals and populations and effective production of new-generation vaccines are essential to tackling future infections (2, 3). As for other infectious viral or bacterial diseases, serological assay kits and vaccines for COVID-19 are generated separately, although their main functional antigenic components are the same. Accordingly, we have established an integrated serological detection and vaccination platform using baculovirus to efficiently combat COVID-19.

Serological testing (antibody testing) determines the antibodies elicited against viral proteins, either from virus infection or vaccination. These antibodies can interact with cognate viral antigen proteins, so they can also be captured by the recombinant antigens in a serological test system. For coronavirus diseases, e.g., SARS and COVID-19, the nucleocapsid (N) and spike (S) viral proteins have been mostly applied as serodiagnostic antigens due to their strong immunogenic activities (4–8). N protein is one of the major structural proteins of coronaviruses and it is expressed abundantly during infection (9, 10). S protein is located on the surface of viral particles and it contains the receptor-binding domain (RBD) that interacts with the host cell-surface angiotensin-converting enzyme 2 (ACE2) receptor (11). Due to this receptor-binding feature, S protein is the primary choice as a vaccine antigen since it can induce the development of neutralizing antibodies. N protein of SARS-CoV-2 also warrants consideration as a target for vaccine development (12–14) as it specializes in stimulating T cell immunity (15) and providing protection in organs such as the spleen and brain (16, 17).

In late 2020, new variants of SARS-CoV-2 possessing the D614G mutation of S became the dominant viruses circulating in humans (18). Emerging SARS-CoV-2 variants that have caused detrimental epidemiological changes and have been designated by the World Health Organization as variants of concern (VOC) (19) include Alpha (lineage B.1.1.7), Beta (lineage B.1.351), Gamma (lineage P.1), and Delta (lineage B.1.617.2) (20–23). Sera from Pfizer BNT162b2 and Moderna mRNA-1273 vaccinees have presented little to no reduction in neutralizing antibody titers against the Alpha and Gamma variants, but exhibited a four- to ten-fold reduction in the titers against pseudovirus bearing mutations of the Beta variant S protein (24–27). Although actual loss of the protective efficacy of current vaccines is still under investigation, these studies highlight the importance of frequent monitoring of the degree of individual or herd immunization and the need to renew vaccine antigens.

Baculovirus (Autographa californica multiple nucleopoly-hedrovirus, AcMNPV) is a biosafety level 1 (BSL-1) (28) insect virus capable of expressing high levels of recombinant proteins with proper protein folding and post-translational modifications (29, 30). Baculovirus expression systems have been shown to strongly express the heavily glycosylated (31) SARS-CoV-2 S protein in studies of protein structure (32, 33), antibody determination (34), and vaccination (35). Baculovirus-expressed N protein of SARS-CoV [which shares 90% amino acid homology with SARS-CoV-2 (12)] exhibited higher sensitivity and specificity in serological detection than that expressed by an *Escherichia coli* system because the former retains its phosphorylation modification (36).

Instead of simply expressing recombinant antigens for purification, baculovirus can “display” a protein or protein fragment on the viral envelope by fusing the protein with baculovirus glycoprotein GP64. We have displayed different fragments of SARS-CoV S protein on the envelope of baculovirus and successfully used the recombinant baculoviruses to precisely identify interleukin induction by SARS-CoV (37). We have also displayed the S of another coronavirus, Porcine Epidemic Diarrhea Virus (PEDV), on the surface of baculovirus and used the recombinant baculovirus as a vaccine to protect piglets from PEDV infection (38). Recombinant antigens displayed on baculovirus are also displayed on the plasma membrane of host insect cells (30), and cells displaying antigens can directly serve as antigen for serological assay (30, 39). Therefore, it is plausible to engineer baculovirus to express SARS-CoV-2 viral antigen on its surface and on insect cells, and then to use the cells and recombinant viruses to establish an integrated platform that provides COVID-19 serum detection and vaccination functions.

**Figure 1:**
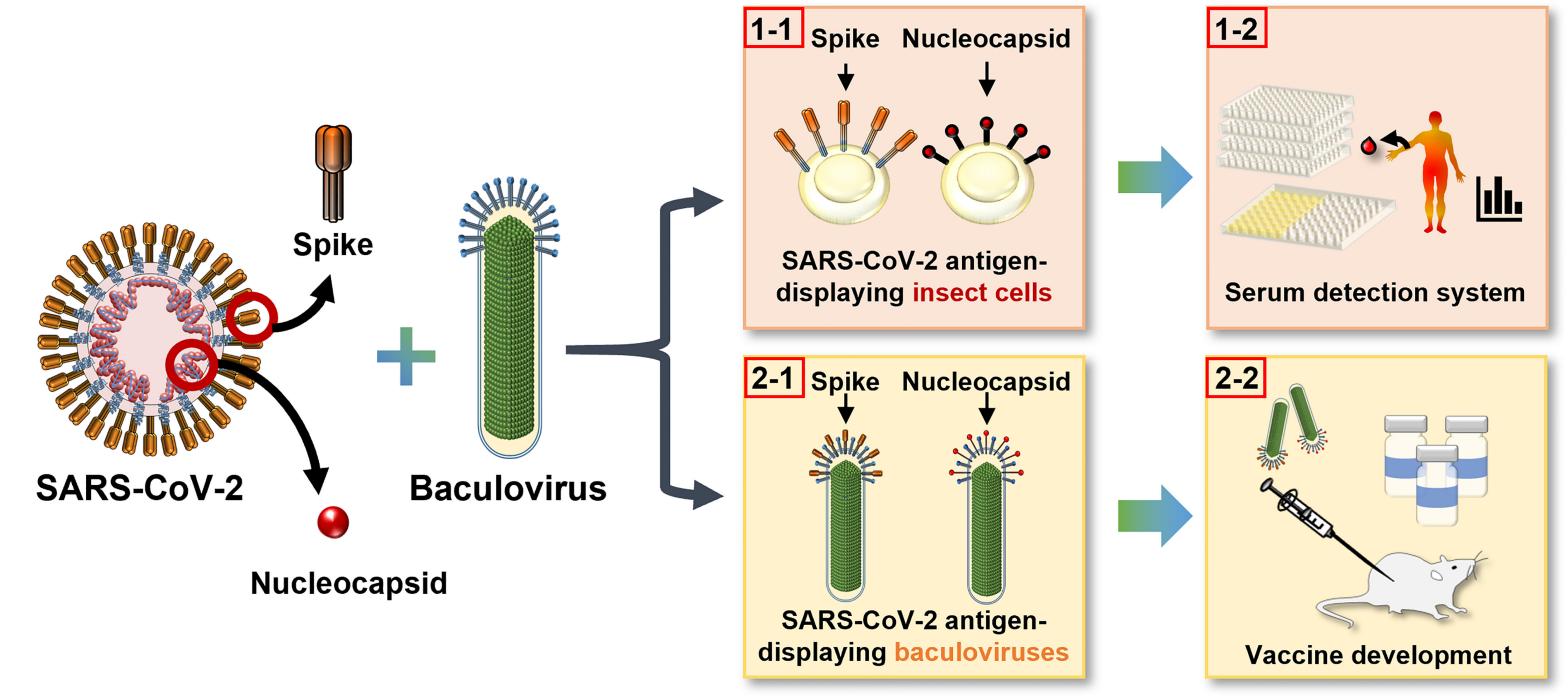
Development of an integrated detection and vaccination system for COVID-19 using baculovirus surface display technology. The S and N proteins of SARS-CoV-2 were displayed on insect cell plasma membrane (1–1) as a convenient and sensitive cell-based ELISA platform for patient serum detection (1-2), and on recombinant baculovirus envelope (2-1) to serve as an effective vector vaccine (2-2).

In this study, we employed the baculovirus expression system to display the N and S proteins from SARS-CoV-2 separately on the surface of insect cell membranes (**Figure 1**, 1-1) and on the recombinant virus envelope (**Figure 1**, 2-1). Insect cells displaying antigen were developed into two cell-based enzyme-linked immunosorbent assays (ELISA) for serological detection of patients’ serum (**Figure 1**, 1-2), and the baculoviruses displaying N or S were used as vector vaccines to tackle SARS-CoV-2 infection (**Figure 1**, 2-2). We examined the feasibility of both cell-based ELISA and baculovirus vaccines and showed that this integrated detection-vaccine platform represents a practical solution for emerging viral diseases.

## Materials and Methods

### Cells and Media

*Spodoptera frugiperda* IPLB-Sf21 (Sf21) cells were cultured at 26°C in TC100 insect medium (Thermo Fisher Scientific, Waltham, MA, USA) supplemented with 10% fetal bovine serum (FBS). HEK-293T cells were cultured in a humidified incubator with 5% CO_2_ at 37°C using Dulbecco’s Modified Eagle’s medium (DMEM) (MilliporeSigma, Burlington, MA, USA) supplemented with 10% FBS and 100 units/mL Penicillin/Streptomycin (Thermo Fisher Scientific, Waltham, MA, USA).

### Construction of Recombinant Baculoviruses

**Figure 2:**
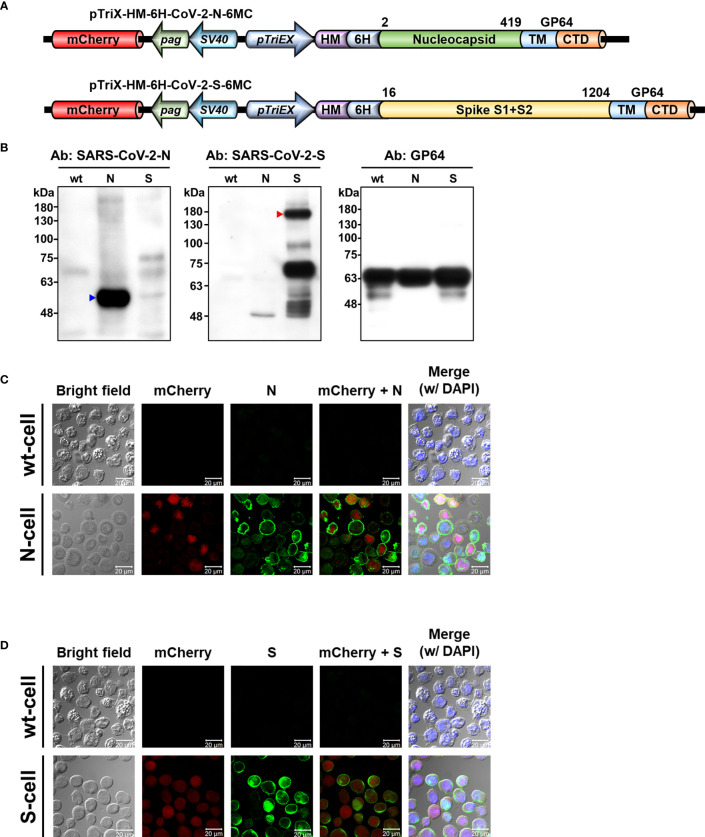
Display of SARS-CoV-2 N and S proteins on baculovirus envelope and insect cell plasma membrane by recombinant baculoviruses. **(A)** Baculovirus expression constructs used for displaying N and S proteins from SARS-CoV-2. The expression constructs pTriEx-HM-6H-CoV-2-N-6MC and pTriEx-HM-6H-CoV-2-S-6MC were used to generate N-Bac and S-Bac baculoviruses, respectively. Numbers indicate amino acid boundaries of N and S from the original sequence. mCherry, mCherry fluorescent protein; *pag*, *pag* promoter; *SV40*, *SV40* promoter; *pTriEX*, pTriEX4 promoter; HM, honeybee melittin signal peptide; 6H, histidine tag; TM, transmembrane domain; CTD, cytoplasmic tailed domain. **(B)** Western blots demonstrating incorporation of N and S proteins in recombinant baculovirus virions. Recombinant N-Bac and S-Bac baculoviruses were purified by sucrose gradient ultracentrifugation and subjected to Western blot using antibodies against the N and S proteins of SARS-CoV-2, as well as GP64 protein of baculovirus. wt: purified wt-baculovirus; N: purified N-Bac; S: purified S-Bac. **(C, D)** Immunofluorescence/confocal microscopy showing the proper display of N **(C)** and S **(D)** on the membrane of insect cells infected with N-Bac or S-Bac, respectively. Immunofluorescence signal was detected using anti-SARS-CoV-2-N **(C)** or anti-SARS-CoV-2-S **(D)** antibodies as the primary antibody, and goat anti-mouse conjugated Alexa Fluor 488 as the secondary antibody. mCherry: red fluorescence expressed from the mCherry reporter gene carried by recombinant viruses. DAPI: nuclear counterstain. wt-cell: Sf21 cells infected with wt-baculovirus. N-cell: Sf21 cells infected with N-Bac. S-cell: Sf21 cells infected with S-Bac. Bar: 20 µm.

The nucleotide sequence of N and S of SARS-CoV-2 isolate Wuhan-Hu-1 (GenBank accession No: MN908947.3) was synthesized with insect codon optimization by Mission BioTec, Taiwan. The full-length sequence of N and the ectodomain of S (amino acid boundaries labeled in **Figure 2A**) were cloned using an In-Fusion^®^ HD Cloning Kit (Clontech Laboratories Inc., Fremont, CA, USA) into pTriEx-4 plasmid (MilliporeSigma, Burlington, MA, USA) under the tripartite promoter *TriEx* comprising the *p10*, *CMV*, and *T7* promoters. A honeybee melittin signal peptide and a hexametric histidine tag were inserted with the promoter and target genes. The transmembrane domain (TM) and cytoplasmic tailed domain (CTD) of GP64 were fused to the C-termini of N and S sequences in the expression constructs. An mCherry gene driven by the binary promoter *SV40-pag* (38) was inserted into the vector as a fluorescence reporter. The resulting pTriEx-HM-6H-CoV-2-N-6MC and pTriEx-HM-6H-CoV-2-S-6MC plasmids were co-transfected with baculoviral DNA FlashBAC™ (Mirus, Madison, WI, USA) into Sf21 cells by Cellfectin (Thermo Fisher Scientific, Waltham, MA, USA) to generate the recombinant baculoviruses N-Bac and S-Bac. After five days of incubation at 26°C, recombinant viruses were harvested from culture supernatants. Single viruses with the highest protein expression were selected by serial dilution and amplified for subsequent experiments.

### Virus Purification

Recombinant and wild-type (wt) baculoviruses were amplified by infecting cells using a multiplicity of infection (MOI) of 0.1. At 5 days post infection, culture supernatants were collected and centrifuged at 10,000 rpm for 30 min to remove cell debris. For each virus, 33 mL supernatant was collected and loaded into 3 mL 25% (w/v) sucrose solution in PBS in an SW28 tube (Beckman, Brea, CA, USA), and then centrifuged at 24,000 rpm and 4°C for 80 min. After decanting the supernatant, the virus pellet was resuspended in Dulbecco’s phosphate-buffered saline (DPBS) (1 mL for sucrose gradient purification or 300 μL for mouse immunization). For pellets subjected to sucrose gradient purification, the virus suspended in DPBS (1 mL) was subjected to a continuous density gradient in SW41 centrifuge tubes (Beckman, Brea, CA, USA) containing 3 mL each of 25%, 40%, and 60% (w/v) sucrose solution. After centrifugation at 28,000 rpm and 4°C for 3 h, the virus particles were collected from the gradient and again subjected to 25% (w/v) sucrose cushion for concentration. Final virus pellets were resuspended in 100 μL DPBS.

### Detecting Recombinant Protein Expression on Baculoviruses and Insect Cells

N-Bac and S-Bac were propagated to determine protein expression in recombinant baculoviruses, and the viral particles were purified by sucrose cushion ultracentrifugation followed by sucrose gradient ultracentrifugation. The purified viruses were mixed with Laemmli Sample Buffer (TAAR-TB2, TOOLS, Taipei, Taiwan) and boiled for 10 min before being subjected to 10% sodium dodecyl sulfate (SDS)-polyacrylamide gel electrophoresis (PAGE). Samples on SDS-PAGE gels were transferred to polyvinylidene fluoride (PVDF) membranes (IPVH00010, MilliporeSigma, Burlington, MA, USA), which were then blocked by 5% skimmed milk in phosphate-buffered saline (PBS) and hybridized with specific antibodies. Anti-SARS-CoV-2-N (GTX135357, GeneTex, Irvine, CA, USA) and Anti-SARS-CoV-2-S (GTX632604, GeneTex, Irvine, CA, USA) antibodies at a 1:5,000 dilution were used to detect the expression of N and S, respectively, on recombinant viruses or infected cells. Expression of GP64 was determined as a loading control using rabbit anti-GP64 (SC65498, Santa Cruz, CA, USA). After incubation with the primary antibody, the membrane was hybridized with secondary antibodies, i.e., horseradish peroxidase (HRP)-conjugated goat anti-mouse IgG (115-035-003, Jackson ImmunoResearch, West Grove, PA, USA) or HRP-conjugated goat anti-rabbit IgG (111-035-003, Jackson ImmunoResearch, West Grove, PA, USA), for signal detection.

To determine the surface display of N and S proteins on insect cells, immunofluorescent staining followed by confocal microscopy was applied. Sf21 cells (1×10^4^) were seeded into 8-well Millicell^®^ EZ slides (PEZGS0816, MilliporeSigma, Burlington, MA, USA) and infected with wt-baculovirus, N-Bac, or S-Bac, in each case using an MOI = 1. Two days after infection, the cells were fixed with 4% paraformaldehyde for 10 min on ice. After blocking with 3% Bovine Serum Albumin (BSA) in DPBS for 1 h at 25°C, the cells were incubated with 1:5,000-diluted anti-SARS-CoV-2-N (GTX135357, GeneTex, Irvine, CA, USA) or anti-SARS-CoV-2-S (GTX632604, GeneTex, Irvine, CA, USA) antibody overnight at 4°C. The cells were washed three times with DPBST (DPBS, plus 0.1% Tween 20) and incubated with 1:200-diluted Alexa Fluor 488 goat anti-mouse IgG secondary antibody (R37120, Thermo Fisher Scientific, Waltham, MA, USA) for 1 h at 25°C. Cell nuclei were counterstained using 49,69-diamidino-2-phenylindole (DAPI) (D1306, Thermo Fisher Scientific, Waltham, MA, USA) at a 1:5,000 dilution for 20 min. After three PBS washes, cells on the slides were sealed with ProLong Diamond Antifade mounting reagent (P36965, Thermo Fisher Scientific, Waltham, MA, USA). All images (1,024 × 1,024 diameter pixels) were acquired using a Zeiss laser confocal microscope (LSM780) with a Fluor 63×/1.40 NA oil-immersion objective. Fluorescence intensity was analyzed using ZEN 2010 software (Zeiss).

### Patients and Blood Samples

The human serum samples used in this study comprised 21 COVID-19 patients, 10 healthy control individuals, and 1 influenza B patient. The 21 COVID-19 patients were all aged over 20 years and had been confirmed as infected with SARS-CoV-2 *via* real-timereverse transcription-polymerase chain reaction (RT-PCR) between February 2020 and May 2021. Serum samples No. 1-11 and No. 19-21 were collected during patient hospitalization at the Tri-Service General Hospital, whereas samples No. 12-18 were collected when patients revisited. The study was approved by the Institutional Review Board (IRB) of Tri-Service General Hospital (No. C202005067), and written informed consent was obtained from all participating patients.

The 10 serum samples utilized as negative controls were taken from the REVEAL Study. This is a community-based cohort study that recruited 23,820 residents from seven townships in Taiwan who were aged 30-65 years in 1991-1992, and from whom blood samples were collected. We randomly selected 10 serum samples from the participant pool who were seronegative for hepatitis B surface antigen and antibodies against hepatitis C virus. The year of sample collection ranged from 1993 to 2005. All participants gave informed consent to participate in the REVEAL Study, and the data collection procedures were reviewed and approved by the Human Subject Research Ethics Committee of Academia Sinica, Taiwan (No. AS-IRB01-08014).

The serum sample from the influenza B patient was collected with informed consent on Day 10 of influenza B virus infection and following approval by the IRB of Tri-Service General Hospital (No. C202005113).

### Western Blotting Analysis to Detect Anti-N and Anti-S Antibodies in Patient Sera

Sf21 cells were infected with wt-baculovirus, N-Bac, or S-Bac at an MOI of 1. The cells were harvested two days after infection by lysing in the sample buffer. After boiling for 10 min, the samples were resolved in a gradient SDS-PAGE gel (HR gradient gel solution, TOOLS, Taipei, Taiwan). Samples on gels were transferred to PVDF membranes, blocked by 5% skimmed milk, and hybridized with anti-His-tagged antibody (GTX628914, GeneTex, Irvine, CA, USA) at a dilution of 1:5,000 or with serum sample at a dilution of 1:5,000. GAPDH expression was determined as a loading control using rabbit anti-GAPDH (GTX100118, GeneTex, Irvine, CA, USA). The membrane was hybridized with secondary antibodies—HRP-conjugated goat anti-mouse IgG (1:5,000 dilution, 115-035-003, Jackson ImmunoResearch, West Grove, PA, USA), HRP-conjugated goat anti-human IgG (1:5,000 dilution, 109-035-097, Jackson ImmunoResearch, West Grove, PA, USA) or HRP-conjugated goat anti-rabbit IgG (1:5,000 dilution, 111-035-003, Jackson ImmunoResearch, West Grove, PA, USA)—for signal detection.

### Cell-Based ELISA

Sf21 cells were infected with wt-baculovirus, N-Bac, or S-Bac at an MOI of 1. The cells were collected two days after the infection of recombinant baculoviruses. After culture media were removed, the cells were washed once with DPBS, seeded into the wells of a 96-well plate (2 × 10^4^ cells/well), and then fixed by 4% paraformaldehyde. After fixation, the cells were incubated with sample blocking buffer (9068, ChonBlock, Chondrex Inc., Woodinville, WA, USA) for 1 h at 25°C. Each serum sample was diluted in sample blocking buffer (1:200) and added to the cell samples and incubated for 2 h at 25°C. After three washes with PBST, HRP-conjugated goat anti-human IgG (1:5,000, 109-035-097, Jackson ImmunoResearch, West Grove, PA, USA) diluted in secondary antibody dilution buffer (90681, ChonBlock, Chondrex Inc., Woodinville, WA, USA) was added to each well. After incubating secondary antibodies for 1 h, the cells were washed three times with PBST and then 3, 3’, 5, 5’-tetramethylbenzidine (TMB) substrate (TMBS-1000-01, Surmodics, MN, USA) was added for color development. Coloring reactions were stopped using 2M sulfuric acid. ELISA absorbance was measured at 450 nm. Each serum sample was individually reacted with cells infected with wt-baculovirus (wt-cells), N-Bac (N-Cells), or S-Bac (S-Cells), with three replicates of each. Mean optical density (OD) of wt-cells was subtracted from the values for N-Cells and S-Cells to represent the final ELISA value of each serum sample.

To assess the specificity of our cell-based ELISA, serum samples were replaced with serum from an influenza B patient or by using the following primary antibodies for ELISA determination: SARS-CoV-2-N polyclonal antibody (pAb) (GTX135357, GeneTex, Irvine, CA, USA), SARS-CoV-2-S monoclonal antibody (mAb) (GTX632604, GeneTex, Irvine, CA, USA), OC43-S pAb (MBS1493076, MyBioSource, San Diego, CA, USA), HKU1-S pAb (40021-RP01, Sino Biological Inc., Beijing, P.R. China), MERS-N pAb (40068-RP01, Sino Biological Inc., Beijing, P.R. China), MERS-S pAb (40069-T62, Sino Biological Inc., Beijing, P.R. China), influenza H3 virus hemagglutinin pAb (representative of influenza A virus antibody) (11707-RP01, Sino Biological Inc., Beijing, P.R. China), influenza B virus hemagglutinin pAb (GTX128542, GeneTex, Irvine, CA, USA), Epstein-Barr virus glycoprotein 350 pAb (40373-RP01, Sino Biological Inc., Beijing, P.R. China), human respiratory syncytial virus type A rsb1734 strain glycoprotein pAb (13029-T52, Sino Biological Inc., Beijing, P.R. China), human respiratory syncytial virus B1 strain glycoprotein mAb (11070-MM15, Sino Biological Inc., Beijing, P.R. China), adenovirus hexon protein mAb (MAB8757, Abnova, Taipei, Taiwan), mAb against pan parainfluenza viruses (MBS602526, MyBioSource, San Diego, CA, USA), and rhinovirus outer capsid protein VP3 mAb (35-703, ProSci, Fort Collins, CO, USA). The influenza B patient serum and primary antibodies were serially diluted two-fold from a starting concentration of 1:100 (for the influenza B patient serum) or 100 µg/ml (for primary antibodies). The secondary antibody for the influenza B patient serum was HRP-conjugated goat anti-human IgG (1:5,000 dilution, 109-035-097, Jackson ImmunoResearch, West Grove, PA, USA), and for primary antibodies it was HRP-conjugated goat anti-mouse IgG (115-035-003, Jackson ImmunoResearch, West Grove, PA, USA) or HRP-conjugated goat anti-rabbit IgG (111-035-003, Jackson ImmunoResearch, West Grove, PA, USA), depending on the primary antibody species applied.

### Protein-Based ELISA

Four different commercialized protein-based ELISA kits were used to compare detection sensitivity with our cell-based ELISAs, namely N-protein-based ELISA kit-1 (Abclonal, Catalog No. RK04139, Woburn, MA, USA), N-protein-based ELISA kit-2 (Proteintech, Catalog No. KE30001, Rosemont, IL, USA), S1-protein-based ELISA kit (Abclonal, Catalog No. RK04138, Woburn, MA, USA), and RBD-protein-based ELISA kit (Proteintech, Catalog No. KE30003, Rosemont, IL, USA). Determination by each kit was performed according to the manufacturer’s procedures with slight modification. Pre-blocked plates in the kits were washed five times with PBST. Each serum sample was diluted in sample blocking buffer (9068, ChonBlock, Chondrex Inc., Woodinville, WA, USA) at a 1:200 dilution and added to the plates for 2 h. The plates were then washed three times with 300 μL per well of 0.1% PBST and incubated with 100 μL HRP-conjugated goat anti-human IgG (1:5,000, 109-035-097, Jackson ImmunoResearch, West Grove, PA, USA) diluted in secondary antibody dilution buffer (90681, ChonBlock, Chondrex Inc., Woodinville, WA, USA) for 1 h. Plates were washed three times with 0.1% PBST, and then TMB substrate (TMBS-1000-01, Surmodics, MN, USA) was added for color development. Coloring reactions were stopped by adding 2M sulfuric acid. The plates were read at an optical density of 450 nm (OD_450_) using an EnSpire multilabel plate reader (PerkinElmer).

### Mouse Vaccination

To amplify the virus for mouse immunization, we infected cells in T75 flasks (1.5 × 10^7^/flask) with N-Bac, S-Bac, or EG-Bac (baculovirus expressing green fluorescent protein) (37) at an MOI of 0.1. At 5 days post infection, culture supernatants were collected and clarified by centrifugation at 10,000 rpm for 30 min. Viral titer in the supernatant was checked by qPCR (with a standard curve established using viruses of known titer, as determined by the 50% tissue culture infectious dose (TCID_50_) method). To concentrate the virus, 33 mL virus supernatant was loaded into a 3 mL 25% (w/v) sucrose solution in an SW28 tube (Beckman, Brea, CA, USA) for centrifugation (24,000 rpm, 4°C, 80 min). After discarding the supernatant, each virus pellet was resuspended in 300 μL DPBS and viral titer was determined again by qPCR. Each virus was dissolved precisely in DPBS to 10^9^ plaque-forming units (pfu)/100 μL for mouse immunization. In general, 20-30 mL of culture (i.e., 2-3 T75 flasks) constituted the final 10^9^ pfu of the virus. Six-week-old inbred female BALB/c mice were purchased from the Taiwan National Laboratory Animal Center. Mice were immunized intramuscularly or intraperitoneally with N-Bac (n=10), S-Bac (n=10), or EG-Bac (n=5). Another five mice were immunized with DPBS as negative controls. Two booster shots were administered at weeks 2 and 4 after the primary immunization. Sera collected from the immunized mice at weeks 4 and 6 were used to determine anti-N and anti-S1 antibody levels by means of 2019-nCoV Nucleocapsid Protein IgG Antibody ELISA Kit (RK04139, Abclonal, Woburn, MA, USA) and 2019-nCoV Spike S1 Protein IgG Antibody ELISA Kit (RK04138, Abclonal, Woburn, MA, USA), respectively, following the manufacturer’s protocols. The five mouse sera (from week 6) displaying the highest anti-S1 contents were subjected to the neutralization assay. Experimental procedures were approved by the Institutional Animal Care and Use Committee (IACUC) of Academia Sinica, Taiwan.

### SARS-CoV-2 Pseudovirus Neutralization Assay

The pseudotyped SARS-CoV-2 virus we used for neutralization assay is a lentivirus carrying the SARS-CoV-2 S gene and a defective HIV-1 genome encoding the luciferase reporter. The recombinant lentivirus was generated by transiently transfecting HEK-293T cells with plasmids pCMV-ΔR8.91, pLAS2w.Fluc.Ppuro, and pcDNA3.1-nCoV-SΔ18 using TransITR-LT1 transfection reagent (Mirus, Madison, WI, USA). Culture media were refreshed at 16 h and harvested at 72 h post-transfection. The supernatant was clarified by centrifugation at 4,000 × *g* for 10 min to remove the cell debris and by passing it through a 0.45-μm syringe filter (Pall Corporation, Port Washington, NY, USA). Heat-inactivated sera (30 min at 56°C) were serially diluted from 1:40 to 1:5,120 and then incubated with 1,000 transducing units (TU) of SARS-CoV-2 pseudotyped lentivirus in DMEM (supplemented with 1% FBS and 100 units/mL Penicillin/Streptomycin) for 1 h at 37°C. The mixture was then inoculated into 1 × 10^4^ HEK-293T cells stably expressing the human ACE2 gene in 96-well plates. Culture media were refreshed at 16 h post-infection, and the cells were continuously cultured for another 48 h. The expression level of the luciferase gene in the cells was determined using the Bright-Glo™ Luciferase Assay System (Promega), and relative light units (RLU) were detected using the Tecan i-control system (Infinite 500). The percentage of inhibition was calculated as the ratio of RLU reduction to the RLU of control (no serum) with the following formula:


$$(\text{RL}{\text{U}}^{\text{Control}}-\text{RL}{\text{U}}^{\text{Serum}})/\text{RL}{\text{U}}^{\text{Control}}\times 100\%$$

Inhibition values below zero were considered background and are shown as zero.

### Statistical Analyses

For cell-based ELISA, indirect ELISA, and SARS-CoV-2 pseudovirus neutralization assay, at least three replicates were conducted for each serum sample. All quantitative data are shown as means ± standard deviations (SD) (error bars). For two-group comparisons, a Mann–Whitney test was performed using GraphPad Prism 9 (GraphPad Software Inc.).

## Results

### Construction of Recombinant Baculoviruses for Surface Display of SARS-CoV-2 Antigens

We generated two recombinant baculoviruses, i.e., N-Bac and S-Bac, to display the N and S proteins of SARS-CoV-2, respectively. The TM and CTD of GP64, the surface glycoprotein of baculovirus, were fused to the C-termini of full-length N and the S ectodomain (**Figure 2A**). These recombinant antigens could then be secreted and anchored on the plasma membrane of insect cells or the baculovirus envelope by the TM of GP64. Indeed, we detected recombinant N and S in purified N-Bac and S-Bac baculoviruses (**Figure 2B**), as well as on the surface of insect cells infected by N-Bac and S-Bac (designated as N-cells or S-cells, respectively), but not the cells infected by wt AcMNPV (wt-cells) (**Figures 2C, D**).

### Surface Display of Antigens on Insect Cells for Serum Analyses

**Figure 3:**
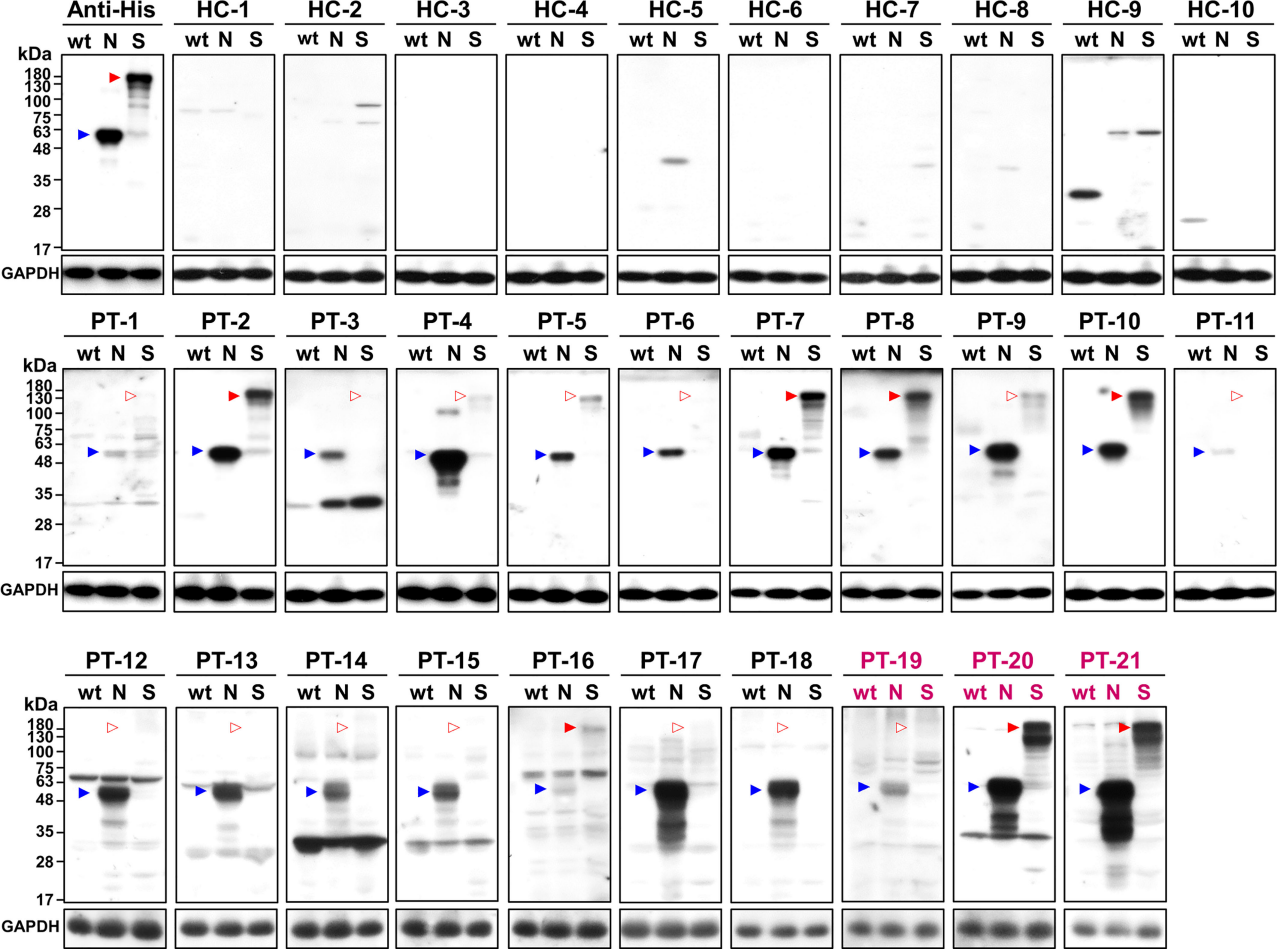
Western blot analysis of healthy controls (HC) and patients (PT) using wt-cells (wt), N-cells (N), or S-cells (S). Arrowheads indicate the position of N (blue) and S (red) proteins. Empty arrowheads indicate positions with weak or lacking apparent signal. Patient sera marked in magenta are samples of the Alpha variant.

To test if N-cells and S-cells could be used as antigens for serum antibody determination, we collected sera from 21 RT-PCR-confirmed COVID-19 patients in Taiwan during their acute or convalescent phases. Most of the samples were from patients that were infected with the early circulating wt virus, but three samples were from patients infected with the more recently emerged Alpha variant (**Table 1**). N-cells and S-cells were first employed in a Western blotting analysis to determine anti-N and anti-S antibody levels in the serum. Compared to 10 healthy control patients that showed no sign of these antibodies, we detected anti-N antibodies in all sera taken from infected patients (although some presented relatively weak signals) (**Figure 3**, blue arrowheads). In contrast, anti-S antibodies were only clearly detected in the sera of seven infected patients (PT-2, 7, 8, 10, 16, 20, and 21), being either weak or absent in the remaining seven samples (**Figure 3**, empty red arrowheads).

**Table 1:** COVID-19 patient data.

Patient no.	Age	Gender	Days post symptom onset upon sample being drawn	Infection stage	Infected virus strain	Symptoms
1	23	M	17	Acute	Wild type	Fever, cough, rhinorrhea
2	41	F	30	Acute	Wild type	Fever, cough, rhinorrhea, headache, sore throat, diarrhea, chest pain
3	23	F	13	Acute	Wild type	Distorted sense of smell, rhinorrhea
4	54	M	21	Acute	Wild type	Cough, sore throat, diarrhea
5	33	M	38	Acute	Wild type	Fever, cough, headache, sore throat, diarrhea, chest pain, dyspnea, chills, nausea
6	21	F	22	Acute	Wild type	Fever, cough, distorted sense of taste and smell, headache, myalgia, rhinorrhea, sore throat, diarrhea, chest pain, dyspnea, chills, nausea
7	21	F	34	Acute	Wild type	Fever, cough, distorted sense of taste and smell, diarrhea, chest pain, dyspnea
8	64	M	32	Acute	Wild type	Fever, cough, rhinorrhea, diarrhea
9	34	M	43	Acute	Wild type	Cough, distorted sense of taste, distorted sense of smell
10	50	F	29	Acute	Wild type	Fever, chills, distorted sense of taste
11	28	F	59	Acute	Wild type	Cough, rhinorrhea, distorted sense of smell, diarrhea
12	80	M	126	Convalescent	Wild type	Fever, cough, rhinorrhea, short of breath
13	54	M	51	Convalescent	Wild type	Myalgia, distorted sense of smell and taste
14	23	F	119	Convalescent	Wild type	Distorted sense of smell, rhinorrhea
15	27	F	204	Convalescent	Wild type	Fever, cough, distorted sense of taste, distorted sense of smell, rhinorrhea
16	66	M	263	Convalescent	Wild type	Fever, cough, rhinorrhea
17	67	F	70	Convalescent	Wild type	Fever, cough, chest pain, short of breath
18	41	F	136	Convalescent	Wild type	Fever, cough, rhinorrhea, headache, sore throat, diarrhea, chest pain
19	43	M	28	Acute	Alpha	No symptoms
20	65	M	32	Acute	Alpha	Fever, short of breath
21	57	M	8	Acute	Alpha	Fever, short of breath

**Figure 4:**
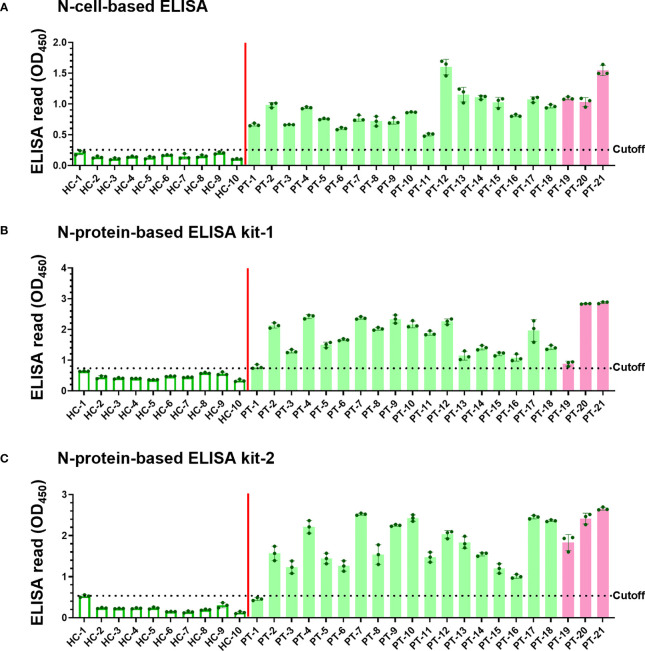
Detection of SARS-CoV-2 infection using our N-cell-based ELISA and comparison to protein-based ELISA kits. Serum samples (1:200 dilution) of 10 healthy controls (HC) and 21 patients (PT) were subjected to N-cell-based ELISA **(A)**, N-protein-based ELISA kit-1 **(B)**, and N-protein-based ELISA kit-2 **(C)** to determine levels of anti-N antibodies. Pink bars represent the three samples from patients infected with the Alpha variant. Cell-based ELISA reads have been normalized to the read derived from individual serum interacting with wt-cells. Dotted line: cutoff value using the mean + 3SD of HC samples.

**Figure 5:**
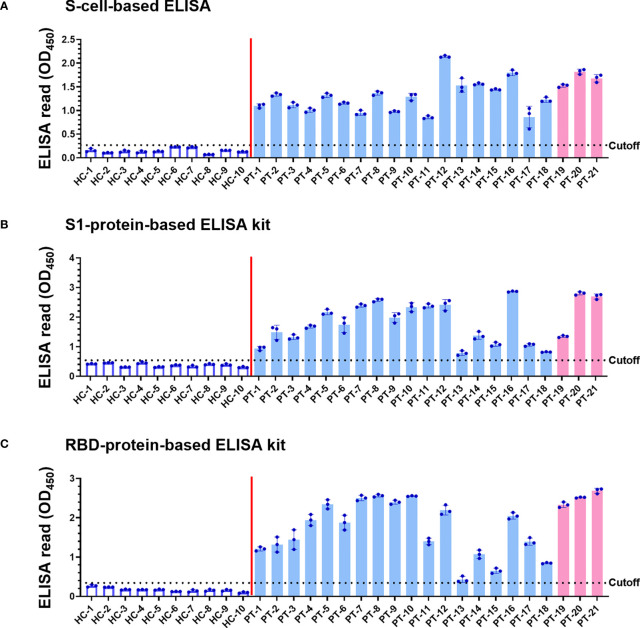
Detection of SARS-CoV-2 infection using our S-cell-based ELISA and comparison to protein-based ELISA kits. Serum samples (1:200 dilution) of 10 healthy controls (HC) and 21 patients (PT) were subjected to S-cell-based ELISA **(A)**, an S1-protein-based ELISA kit **(B)**, and an RBD-protein-based ELISA kit **(C)** to determine levels of anti-S antibodies. Pink bars represent the three samples from patients infected with the Alpha variant. Cell-based ELISA reads have been normalized to the read derived from individual serum interacting with wt-cells. Dotted line: cutoff value using the mean + 3SD of HC samples.

Since the denaturing Western blot analysis may overlook conformation-dependent antibodies, we developed a novel cell-based ELISA to determine the possible existence of conformational antibodies in the patient sera. N-cells, S-cells, and wt-cells were directly seeded into 96-well plates. Each serum sample was diluted at a 1:200 dilution and added into the well of micro-well plates to interact with these cells in parallel. After washing away non-specific serum antibodies that do not interact with the antigen, we added HRP-conjugated anti-human IgG to recognize the specific human serum antibodies binding on the S and N antigens. Human IgG levels were then revealed by color development upon adding chromogenic HRP substrate and quantified by spectrophotometry. To eliminate background signal developed by insect cells or wt-baculovirus infection, the absorbance of wt-cells was subtracted from those of N-cells and S-cells to represent final ELISA values (**Figures 4A**, **5A**). Absorbances prior to subtracting the signal of wt-cells are shown in **Supplementary Figure 1**. To compare sensitivity between our cell-based ELISA and conventional ELISA using purified proteins, we selected four ELISA kits deploying different SARS-CoV-2 antigens to analyze the same serum samples in parallel (**Figures 4B, C**, **5B, C**). The mean ELISA value established from all healthy controls plus three SD was set as the cutoff for each ELISA system (**Figures 4** and **5**, dotted lines). Test subjects with anti-S or anti-N antibody levels exceeding the cut-off value were considered to have a positive reaction.

The sera of all 21 patients tested positive in ELISAs using both N-cells (**Figure 4A**) and S-cells (**Figure 5A**). Notably, samples that had presented low signals on Western blots were strongly detected by both N-cell- and S-cell-based ELISAs. In contrast, some protein-based ELISA kits gave relatively low signals for those samples, and the N-protein-based ELISA kit-2 even failed to detect one positive sample (**Figure 4C**, PT-1). This trait was also revealed by the positive/negative (P/N) ratio (**Tables 2** and **3**), which was calculated by dividing the signal for individual patients by the average for the healthy controls. Our N-cell-based ELISA showed P/N ratios >3 for all patient serum samples, whereas the two N-protein-based ELISAs presented low values for some of the patient samples (**Table 2**). For S-based ELISAs, our S-cell-based ELISA displayed P/N ratios higher on average than those of an S1-protein-based ELISA kit and slightly lower than those of an RBD-protein-based ELISA kit (**Table 3**). Notably, some patient samples presenting a relatively low P/N ratio for the S1-protein-based and RBD-protein-based ELISA kits showed high P/N ratios under our S-cell-based ELISA (**Table 3**, PT-13, PT-14, PT-15, and PT-18).

**Table 2:** P/N ratios calculated from three N-based ELISAs applied to 21 patient samples.

PT No.	N-cell-based ELISA	N-protein-based ELISA kit-1	N-protein-based ELISA kit-2
1	4.411 ± 0.150	1.690 ± 0.142	1.923 ± 0.152
2	6.548 ± 0.267	4.576 ± 0.183	6.604 ± 0.734
3	4.415 ± 0.030	2.770 ± 0.114	5.194 ± 0.639
4	6.249 ± 0.103	5.193 ± 0.137	9.329 ± 0.649
5	5.030 ± 0.077	3.219 ± 0.191	6.081 ± 0.536
6	4.000 ± 0.119	3.576 ± 0.066	5.312 ± 0.529
7	5.097 ± 0.315	5.100 ± 0.087	10.632 ± 0.105
8	4.788 ± 0.526	4.342 ± 0.098	6.482 ± 1.004
9	4.801 ± 0.330	5.025 ± 0.287	9.489 ± 0.097
10	5.762 ± 0.049	4.649 ± 0.234	10.244 ± 0.329
11	3.343 ± 0.148	4.033 ± 0.148	6.223 ± 0.512
12	10.653 ± 0.837	4.846 ± 0.198	8.564 ± 0.392
13	7.671 ± 0.788	2.456 ± 0.308	7.716 ± 0.600
14	7.372 ± 0.209	3.023 ± 0.156	6.527 ± 0.185
15	6.817 ± 0.558	2.588 ± 0.127	5.057 ± 0.489
16	5.398 ± 0.123	2.317 ± 0.246	4.248 ± 0.197
17	7.143 ± 0.298	4.221 ± 0.761	10.337 ± 0.180
18	6.406 ± 0.184	3.036 ± 0.148	9.971 ± 0.089
19	7.267 ± 0.150	1.905 ± 0.168	7.718 ± 0.830
20	6.861 ± 0.484	6.097 ± 0.021	10.188 ± 0.554
21	10.284 ± 0.558	6.179 ± 0.066	11.197 ± 0.151

P/N ratio value: 
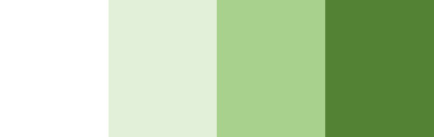
       0 3 6 9 12

**Table 3:** P/N ratios calculated from three S-based ELISAs applied to 21 patient samples.

PT No.	S-cell-base ELISA	S1-protein-based ELISA kit	RBD-protein-based ELISA kit
1	7.428 ± 0.292	2.497 ± 0.174	7.089 ± 0.305
2	9.052 ± 0.222	3.947 ± 0.630	7.744 ± 1.119
3	7.547 ± 0.395	3.510 ± 0.214	8.481 ± 1.462
4	6.805 ± 0.303	4.460 ± 0.139	11.406 ± 0.834
5	8.944 ± 0.296	5.747 ± 0.243	13.755 ± 0.687
6	7.818 ± 0.153	4.624 ± 0.696	11.021 ± 1.095
7	6.424 ± 0.339	6.343 ± 0.141	14.704 ± 0.402
8	9.249 ± 0.289	6.836 ± 0.138	15.048 ± 0.194
9	6.606 ± 0.116	5.270 ± 0.438	14.064 ± 0.297
10	8.745 ± 0.502	6.198 ± 0.386	14.989 ± 0.059
11	5.794 ± 0.211	6.312 ± 0.183	8.202 ± 0.485
12	14.488 ± 0.139	6.400 ± 0.479	12.880 ± 0.723
13	10.354 ± 0.981	2.078 ± 0.209	2.535 ± 0.447
14	10.566 ± 0.146	3.646 ± 0.340	6.283 ± 0.629
15	9.768 ± 0.100	2.866 ± 0.177	3.820 ± 0.392
16	12.159 ± 0.389	7.603 ± 0.043	12.033 ± 0.480
17	5.839 ± 1.537	2.822 ± 0.118	8.174 ± 0.523
18	8.290 ± 0.354	2.176 ± 0.030	4.967 ± 0.085
19	10.323 ± 0.220	3.577 ± 0.096	13.693 ± 0.406
20	12.329 ± 0.354	7.436 ± 0.144	14.779 ± 0.070
21	11.370 ± 0.536	7.157 ± 0.231	15.776 ± 0.389

P/N ratio value: 
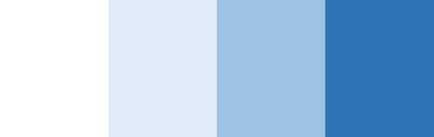
       0  4 8 12 16

**Figure 6:**
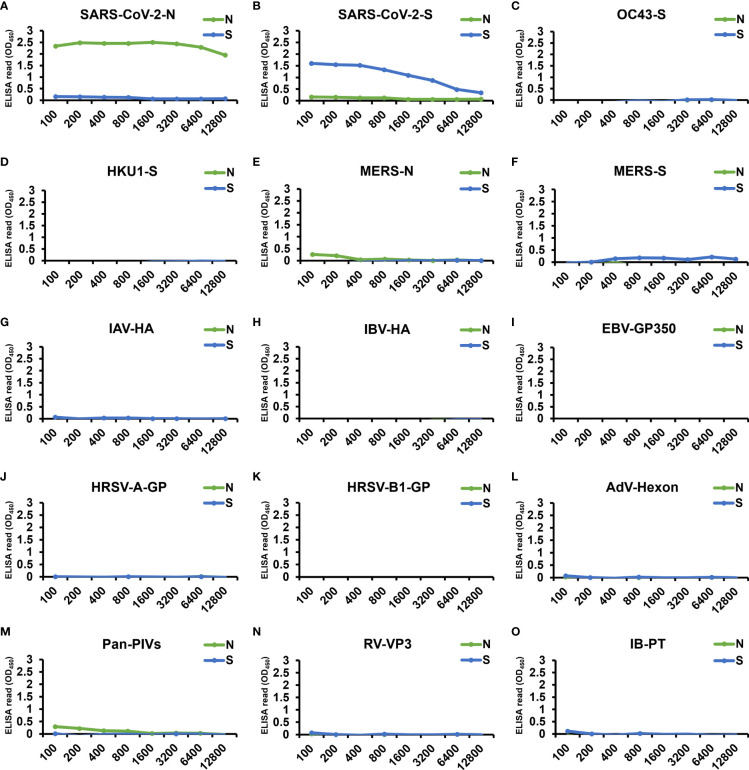
Examination by our cell-based ELISAs of the cross-reactivity of antibodies from other human coronaviruses and viruses causing upper respiratory tract infection. The specificity of our cell-based ELISAs using N-cells (green) or S-cells (blue) was tested by applying 9 pAb, 5 mAb, and 1 patient serum. These include positive control antibodies, i.e., **(A)** SARS-CoV-2-N pAb and **(B)** SARS-CoV-2 S mAb, antibodies against other human coronavirus antigens, i.e., **(C)** OC43-S pAb, **(D)** HKU1-S pAb, **(E)** MERS-N pAb, and **(F)** MERS-S pAb, and antibodies specific to the antigen of viruses causing upper respiratory tract infections: **(G)** influenza A virus hemagglutinin (IAV-HA) pAb, **(H)** influenza B virus hemagglutinin (IBV-HA) pAb, **(I)** Epstein-Barr virus glycoprotein 350 (EBV-GP350) pAb, **(J)** human respiratory syncytial virus type A rsb1734 strain glycoprotein (HRSV-A-GP) pAb, **(K)** human respiratory syncytial virus B1 strain glycoprotein (HRSV-B1-GP) mAb, **(L)** adenovirus hexon protein (AdV-Hexon) mAb, **(M)** pan parainfluenza viruses (Pan-PIVs) mAb, and **(N)** rhinovirus outer capsid protein VP3 (RV-VP3) mAb. All antibodies were serially diluted from a starting concentration of 100 µg/mL. **(O)** A serum sample from an influenza B patient was also assessed (IB-PT). Each experiment was performed once. X-axes, antibody dilution factor.

To verify the specificity of the two cell-based ELISAs, we applied 9 pAb and 5 mAb from other human coronaviruses or viruses causing upper respiratory tract infections, as well as serum from an influenza patient, to the two systems (**Figure 6**). Only the antibodies specific to SARS-CoV-2-N (**Figure 6A**) and SARS-CoV-2-S (**Figure 6B**) generated significant ELISA signal upon interacting with our N- and S-cell-based ELISA, respectively, whereas the other antibodies did not exhibit signal in either cell-based ELISA (**Figures 6C–O**), proving that the signals detected from COVID-19 patients were explicitly derived from the interaction of serum antibodies generated by SARS-CoV-2 infection.

### Surface Display of Antigens on Baculovirus as Potential Vector Vaccines

**Figure 7:**
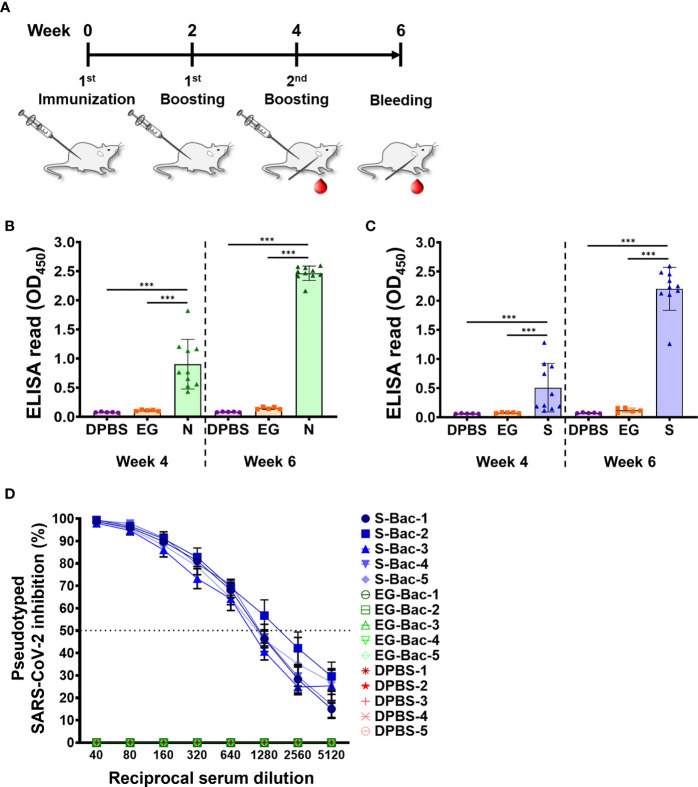
N-Bac and S-Bac as potential COVID-19 vaccines. **(A)** Immunization schedule of N-Bac and S-Bac in mice. Serum samples of each mouse were collected at weeks four and six after primary immunization. **(B, C)** Indirect ELISA against purified N **(B)** or S1 **(C)** to determine the levels of specific anti-N and anti-S1 IgG antibody in mice immunized with DPBS (n=5), EG-Bac (n=5), N-Bac (n=10), or S-Bac (n=10). Mann–Whitney test: *P< 0.05; **P<0.01; ***P<0.001. **(D)** Neutralizing activities of mouse sera (week six, n=5) against SARS-CoV-2 pseudovirus. Percent inhibition of pseudovirus infection of HEK293T cells was determined relative to the virus without serum addition. Mean values ± SD from three independent experiments are shown.

To evaluate the potential of using N-Bac and S-Bac as vaccine antigens, we immunized mice with the recombinant baculoviruses (**Figure 7A**) and then examined the antibodies generated in mouse sera that are specific to S and N proteins. In contrast to mice immunized with DPBS or baculovirus expressing green fluorescent protein (EG-Bac), mice immunized with N-Bac or S-Bac developed high levels of antibodies recognizing the N and S1 antigens, respectively (**Figures 7B, C**). In a serial dilution test, sera from most of the mice injected with N-Bac or S-Bac presented strong ELISA signals even at the highest dilution (1:40,000) compared with those from mice immunized with DPBS or EG-Bac (**Supplementary Figure 2**), supporting the strong immunogenicity of baculovirus vaccines. We adopted a neutralization test with lentivirus-based SARS-CoV-2 pseudovirus to test the infection inhibitory effect of mouse antisera. As determined by serial dilution, the sera of mice immunized with S-Bac inhibited pseudovirus infection in HEK293T cells, with 50% inhibition achieved at a dilution of 640-1,280-fold (**Figure 7D**). Antisera from N-Bac immunization did not inhibit infectivity since the pseudovirus only harbors S protein from SARS-CoV-2. These results demonstrate that N-Bac and S-Bac are antigenic and that the antibodies induced by S-Bac effectively neutralize the function of SARS-CoV-2 S protein.

## Discussion

COVID-19 has spread globally, so the development of serological diagnoses to measure immunity and vaccines that provide preventive protection in individuals has become increasingly important. Serological tests that detect antibodies in serum help to diagnose asymptomatic cases in a population and to track a prior infection or contact with infected individuals, and enable assessments of vaccination efficacy (34, 40). The occurrence of mutant variants is further challenging the efficiency of renewing SARS-CoV-2 vaccines. In this study, we developed an integrated detection-vaccine platform comprising antigen-displaying insect cells for serological analyses and antigen-displaying baculovirus as a vector vaccine for immunization.

Since the beginning of the COVID-19 pandemic, numerous serological testing products have been developed (5, 6, 8, 41–43). Generally, the sensitivity of detecting antibodies in serum, especially IgG, reaches ~80% after day 15 of symptom onset (6, 8, 44, 45). S-based antigens (i.e., RBD, S1, or full-length S) were found to be more sensitive than N-based antigens for serum detection (5, 6). Our analysis of ELISA kits using purified proteins as antigens showed that one patient sample (PT-1) was well detected by our N-cell-based ELISA, but it barely passed the cutoff when tested with N-protein-based ELISA kit-1 and it was even discriminated as a false-negative by N-protein-based ELISA kit-2 (**Figure 4**). This discrepancy may be due to different systems used to generate the antigen. Compared with antigens expressed by bacterial systems, the N-antigen expressed by insect cells in cell-based systems preserves post-translational modifications and may increases the sensitivity of detection (36).

In terms of S-based antigens, the RBD-protein-based ELISA kit exhibited slightly superior P/N ratios to those generated by our S-cell-based ELISA, whereas the S1-protein-based ELISA kit exhibited relatively lower ratios (**Table 3**). Since RBD is small and relatively easy to produce, many commercialized ELISAs use RBD as the antigen (6, 8, 45). However, some of the patients’ sera presented a relatively low signal in the RBD-protein-based ELISA kit we tested (**Figure 5C**). Moreover, previous study has shown that using full-length S as antigen may result in better sensitivity than what can be achieved using RBD antigen (5). These outcomes might be due to the fact that full-length S protein contains more epitopes that can potentially interact with antibodies targeting regions other than S1 or RBD. Another possibility is that antibodies against S in these sera primarily recognize the conformational epitopes only presented on the full-length or trimerized S structure. As revealed by our Western blot analysis, many sera indeed contain conformation-dependent anti-S antibodies (**Figure 3**). By applying our cell-surface display methodology, the cell-based ELISA using full-length S can easily be produced without the need for protein purification and it can be applied for more comprehensive serum detection.

Our system is different from the cell-based ELISA systems using monoclonal antibodies to quantify cell surface antigens or determine the degree of viral infection in cells (46–48) in that we employed the antigen on cells to interact directly with crude serum samples. Human antiserum contains complex components and generates strong background signals by cross-reacting with multiple proteins in cells (49). Therefore, although there is a massive demand for patient serum detection and analysis, cell-based systems are not yet used in ELISA to assay the serum from patients. To circumvent the problem, we used insect cells, which are evolutionarily distinct from mammalian cells, to limit cross-reactivity in our cell-based ELISA system. In addition, baculovirus blocks transcription and translation in the infected insect cells (50), thereby further reducing background protein levels. By subtracting the reads of wt-cells, we could further limit ELISA background from insect cells and polystyrene solid phase (49). Consequently, both our newly established N-cell- and S-cell-based ELISAs present high sensitivity and specificity.

Baculoviruses with surface display of antigens have proven to be effective animal vaccines (51–54). In our experiments, both antigen-displaying N-Bac and S-Bac baculoviruses exhibited strong antigenicity, indicating that they can serve as vector vaccines for SARS-CoV-2 vaccination. In general, the production of vector vaccines requires particular efforts to prevent the emergence of recombined and uncharacterized pathogens or the introduction of adventitious agents (55–57). The former is especially critical in cases where viral vector vaccines are being generated using human viruses, e.g., adenovirus and adeno-associated virus. To ensure the viruses are replication-defective in human cells, one or two essential genes are deleted from the vector and they are instead expressed by the host cells during cell culture (58). Therefore, assembly of recombinant viruses needs to be monitored throughout the manufacturing process. In contrast, baculovirus cannot replicate in humans or any other mammals, so such complex assembly processes and monitoring are not necessary.

Vaccines produced by baculovirus vectors present two other advantages. Firstly, they are highly safe for humans and, secondly, they can stimulate particular immune responses upon certain modifications. Unlike other vector vaccines that primarily involve using BSL-2 human-infectious viruses, baculovirus is a safe BSL-1 virus from insects that lacks vertebrate infectivity and does not insert its genome into host cells (59). N-Bac or S-Bac function as combinations of subunit and DNA vaccines as they express and display the antigens. Upon injecting baculovirus vector vaccine, the antigens displayed on the baculoviral surface first engage antigen-presenting cells (APCs) and activate the antigen-specific immune response through the major histocompatibility complex II (MHC-II)-mediated antigen presentation pathway (60, 61). After that, antigens that are endogenously-expressed by baculovirus vectors can be cross-presented *via* MHC-I to APCs and activate T helper type 1 (Th1) cells that stimulate macrophages and cytotoxic T lymphocytes (CTLs) to eradicate the virus-infected cells (62, 63). Such cell-mediated immunity can strongly enhance the efficacy of baculovirus vector vaccines (62, 63). Furthermore, baculovirus viral particles can serve as an adjuvant in mammals, which boosts immune responses during vaccination (64, 65). Therefore, N-Bac and S-Bac could be used without the need for additional adjuvant treatment. In this study, S-Bac, in particular, elicited neutralization antibodies against SARS-CoV-2 pseudovirus infection (**Figure 7**). Previously, N protein vaccination against SARS-CoV was shown to be effective by inducing CTL to destroy infected cells (66), but the effect of N-Bac vaccination needs to be evaluated in appropriate animal models. We are currently undertaking such experiments.

Pre-existing immunity against viral vectors is a crucial issue for human viral vector vaccines as it may reduce vaccine efficacy and increase the time and cost required for vaccine validation before clinical trials (67). In the case of N-Bac and S-Bac, even if the first injection induces immune responses against baculoviruses in an individual, the antigens displayed on the virus may still elicit antigen-specific immunity. In our animal experiments, follow-up immunization enhanced anti-N and anti-S antibody responses (**Figures 7B, C**). Pre-existing immunity did not appear to have a significant inhibitory effect. In addition, previous studies have reported that the core α1,3-fucosylation present in recombinant proteins produced by certain insect cell lines engenders a risk of causing allergic reactions because humans cannot produce α1,3-fucosylated N-glycoproteins (68, 69). However, α1,3-fucosylation only occurs in *Trichoplusia ni* cell lines (e.g., High Five^®^ cells), but not in the Sf9 cell line from *S. frugiperda* or the BmN cell line from *Bombyx mori* (70, 71). Moreover, co-expression of *Pseudomonas aeruginosa* GDP-6-deoxy-D-lyxo-4-hexulose reductase (RMD) or addition of PNGase A in *T. ni* cell lines has been shown to prevent α1,3-fucosylation and reduce allergic reactions (69). By using the Sf21 cell line in this study, we have circumvented the problems of allergenicity in vaccination, which would also be the case for the Sf9 cell line because it is derived from the same insect species.

In terms of the problem of emerging SARS-CoV-2 variants, since our cell-based ELISA system employs both N and S antigen, we hypothesize that a sample undetected by one antigen may be detected by the other. Especially for the S antigen that has been reported to possess multiple amino acid mutations (72), antiserum arising from mutant virus infection may still possess anti-S antibodies that target the conserved epitopes on S, especially on the S2 subunit, and so they can interact with the full-length S antigen in our cell-based ELISA system. Due to the limitations of serum sample collection in Taiwan, we can only test a relatively small number of subjects. In our results, the three Alpha variant samples could be detected by both our N and S detection systems with a similar efficiency to that for samples from the early circulating wt strain (**Figures 4**, **5**, and **Tables 2** and **3**). Moreover, in terms of vaccines, our S-Bac vaccine uses the same spike sequence employed in current vaccines under emergency use authorization (EUA), hence it may exhibit similar degrees of reduced neutralizing antibody production upon encountering mutant virus strains, i.e., its neutralizing antibody titers may decrease upon encountering Beta variants, but not in the case of Alpha and Gamma variants (24, 73). If necessary, S-Bac with mutant spike genes could still be generated to effectively overcome mutant SARS-CoV-2 viruses. Promisingly, since N-protein-based immunization has been shown to enhance immune responses through the induction of CTLs (62, 66, 74) or complement activation (75), co-administration of our N-Bac vaccine may help combat emerging SARS-CoV-2 variants.

In conclusion, we have established a convenient system that integrates two of the most important tasks necessary to combat viral disease, i.e., detection and vaccination. By using baculovirus surface display strategies, large multimeric or membrane proteins (e.g., S) or nucleic acid-associated proteins (e.g., N) can be fully displayed on the cell membrane or baculoviral envelope to maintain proper protein conformation and the epitopes for antibody recognition (in serological tests) or antibody stimulation (as vaccine antigens). This system is not only useful for tackling the COVID-19 pandemic, but it could also be very valuable for effectively combating emerging and recurrent viral diseases in the future, especially for those that are highly infectious or highly toxic and can only be handled in a BSL-3 laboratory.

## Data Availability Statement

The original contributions presented in the study are included in the article/**Supplementary Material**. Further inquiries can be directed to the corresponding authors.

## Ethics Statement

The studies involving human participants were reviewed and approved by the Institutional Review Board (IRB) of Tri-Service General Hospital and Human Subject Research Ethics Committee of Academia Sinica. The patients/participants provided their written informed consent to participate in this study. The animal study was reviewed and approved by Institutional Animal Care and Use Committee (IACUC) of Academia Sinica, Taiwan.

## Author Contributions

S-CW, W-TH, C-HT, and Y-CCha conceived and designed the research. C-HC, F-YC, and H-IY collected data and contributed samples. S-CW, W-TH, H-RL, C-YL, and Y-CCho performed the research. S-CW, W-TH, C-HC, F-YC, H-IY, Y-CCho, C-HT, and Y-CCha analyzed the data. H-IY, Y-CCho, C-HT, and Y-CCha wrote the manuscript. All authors contributed to the article and approved the submitted version.

## Funding

This research was funded by grants MOST 109-2927-I-001-511, MOST 109-2321-B-033-001, MOST 109-2327-B-016-004, MOST 110-2923-B-001-001, and MOST 110-2313-B-001-010 from the Ministry of Science and Technology of Taiwan, ROC; VTA110-A-4-1 from VGH, TSGH, AS Joint Research Program; and grants under the 2021 NBRP Translational Research Project, Academia Sinica, Taiwan, ROC.

## Conflict of Interest

The authors declare that the research was conducted in the absence of any commercial or financial relationships that could be construed as a potential conflict of interest.

## Publisher’s Note

All claims expressed in this article are solely those of the authors and do not necessarily represent those of their affiliated organizations, or those of the publisher, the editors and the reviewers. Any product that may be evaluated in this article, or claim that may be made by its manufacturer, is not guaranteed or endorsed by the publisher.
