# Supplementary material for: An Integrated Platform for Serological Detection and Vaccination of COVID-19
Source: Front Immunol. 2021 Dec 23;12:771011. doi: 10.3389/fimmu.2021.771011 (PMC8734241; doi:10.3389/fimmu.2021.771011)
Supplement: Supplementary file 1 [file DataSheet_1.docx]

Supplementary Material

## Supplementary Figures


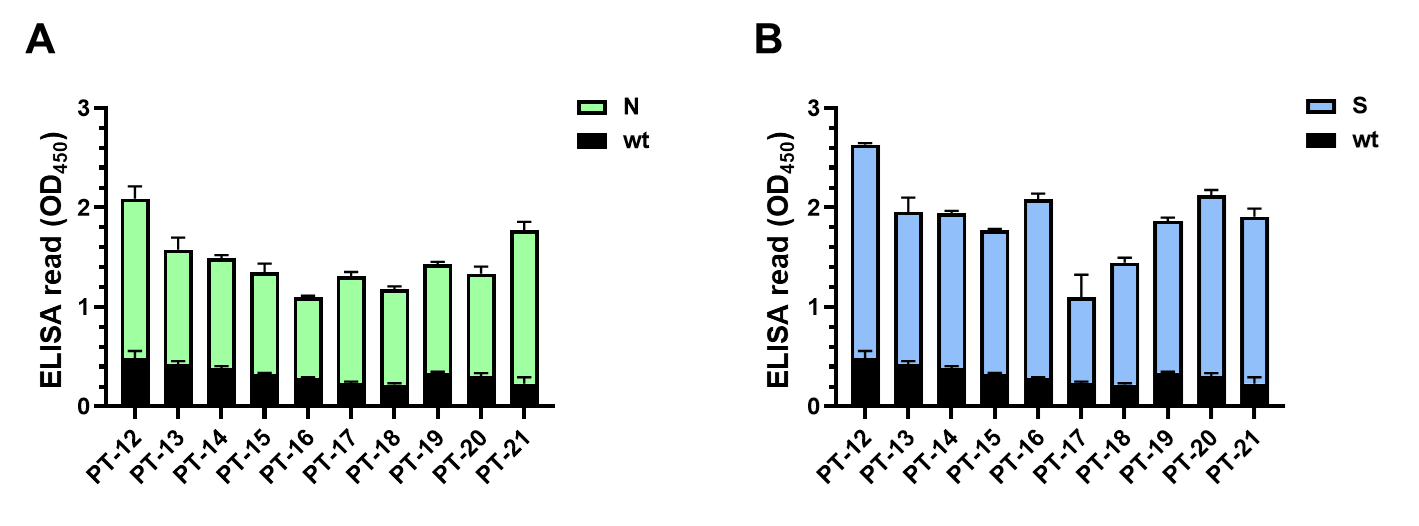


**Supplementary Figure 1. Original ELISA values before subtracting the reads of wt-cells.** Original OD values of the 10 latest-collected sera upon interacting with our N-cell-based ELISA (A) and S-cell-based ELISA (B). Black bars represent the OD values for wt-cells. The final ELISA values presented in Figs. 4 and 5 were derived by subtracting the values for wt-cells (black bars) from those of N-cells/S-cells (green/blue bars). Each bar represents the mean values ± SD (error bar) from three independent experiments.


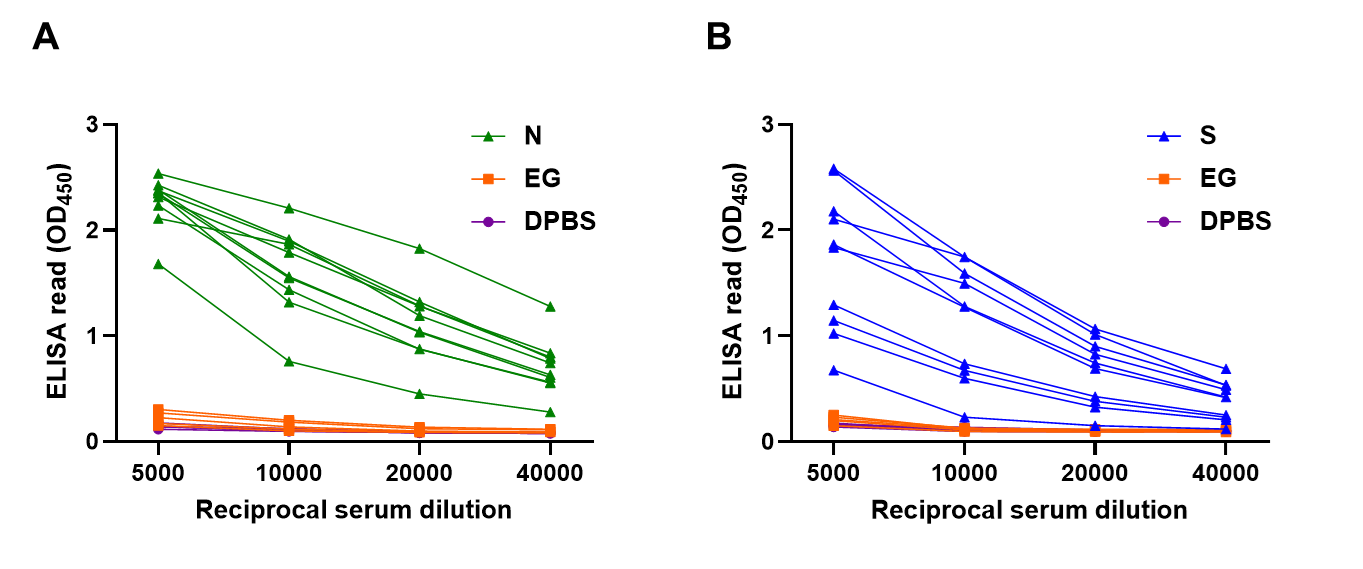


**Supplementary Figure 2.** **Reactivities of mouse sera to N and S1 antigens in indirect ELISAs.** Sera from mice immunized with DPBS (n=5), EG-Bac (n=5), or N-Bac (n=10)/S-Bac (n=10) were two-fold serially diluted from 1:5,000 to 1:40,000 and applied to indirect ELISAs using purified N (A) or S1 (B) as the antigen. Individual lines represent ELISA signals from a given sample in one experiment.
